# An Operational Perspective of The Changing Prosthetics & Orthotics Landscape

**DOI:** 10.33137/cpoj.v4i2.35996

**Published:** 2021-09-21

**Authors:** JM Brandt

**Affiliations:** 1 Ability Prosthetics & Orthotics, 660 West Lincoln Highway, Exton, PA 19341, USA.

**Keywords:** Innovation, Interface, Amputation, Limb Loss, Rehabilitation, Modular, Prosthetic Sockets, Prosthetics, Medical Equipment

## Abstract

Leading the growth of a private prosthetic and orthotic (P&O) practice, as clinician and founder, I developed a unique perspective of this rapidly changing profession. Many positive influences from my early career shaped my vison toward an innovative practice model, as well as the need to elevate the standard of care through education and the use of outcome measures. As the practice model expanded, advancements were made in electronic health records (EHR), best-in-class outsource fabrication, and clinical research. To better support clinicians and patients served, an organizational structure with an executive team was built. The practice model achieved operational efficiency through documenting best practices, developing a hiring and onboarding process, and establishing key performance indicators aligned with quality clinical care. As a regional clinical care organization, the practice model seized an opportunity to reach more patients through a partnership that brought the optimal strategic and cultural fit. Bringing our innovative P&O practice model together with expertise in lean facility design, scanning, fabrication, sensor technology, product development and clinical care experience from around the world, we can advance care standards and improve the patient experience in exciting new ways.

## INTRODUCTION

The landscape of the prosthetic and orthotic (P&O) profession has changed dramatically over the past twenty years, along with the rest of the healthcare sector. For private P&O practices, these changes have represented advancements, challenges, and opportunities. Navigating these changes while continuously striding to provide the highest level of P&O intervention and patient care has required innovative thinking and adopting new perspectives. As a clinician and the founder of a private P&O practice, I have had the opportunity to lead an organization throughout this changing landscape. This has provided me unique experiences, which can inform the P&O profession about the future growth of the private practice sector and further advancement of the standard of care.

## Entry Into The Profession and Influences Toward A P&O Practice Model

In 1993, as a junior at the Pennsylvania State University (PSU), I first volunteered in a regional P&O department to ‘see what the field was all about.’ To my good fortune, I shadowed a Certified Prosthetist Orthotist (CPO), who happened to be one of the American Board for Certification's (ABC) early certifies who also possessed an undergraduate degree, though not required at the time. The profession was in the midst of rapidly changing entry level educational requirements for P&O certificate programs. My early mentor impressed upon me a need to sanctify the clinical care we provide as professionals by requiring advanced education, contributing to building research evidence, and measuring clinical outcomes to both quantify the benefit our P&O interventions provide to our patients in order to demonstrate the value of the care we provide.

Upon graduating from PSU in 1995 with a Bachelor of Science in Psychology, I enrolled in a technical program for P&O fabrication, to further my knowledge and experience in the profession and ultimately pursue a career as an ABC certified practitioner. What felt like a brief phase back then, my time training and working as a technician provided me with valuable insights into the role fabrication played in the P&O practice model at the time and informed my perspective on opportunities for innovative care models for the future.

In 2000, I graduated from the Northwestern University Prosthetic & Orthotic Center with certificates in prosthetics and orthotics. I then embarked on residencies at the Rehabilitation Institute of Chicago (now Shirley Ryan Ability Lab) and AI DuPont Children's Hospital in Delaware. In residency, I continued to develop my clinical skillsets in evaluation and measurement, as well as advance my clinical judgment around treatment decisions and component recommendation. As a newly minted practitioner, proper documentation and justification of my services remained a challenge. At that time, the P&O profession looked for ways to make more evidence-based decisions.

I recall reading an influential article at that time entitled, ‘*Facing the Future of Orthotics and Prosthetics Proactively: Theory and Practice of Outcomes Measures as a Method for Determining Quality of Services*‘, by Andrian Pollack, PhD, MIPEM and Stefan Moser, CPO, CPed.^1^ This article captured the sentiment of the landscape and future direction of the P&O profession in 1997, twenty-four years ago. The authors described the need for the profession to implement routine documentation of objective outcome measures to quantify the quality and evaluate the cost-effectiveness to secure the future success of the P&O profession. Little did I know at that time that the message from these authors would serve to guide decisions I would make almost two decades later.

## Expanding A New P&O Practice Model Through Growth and Research

In 2004, I founded Ability Prosthetics & Orthotics with a guiding mission of 1) providing patients with the most appropriate, affordable, and technologically advanced devices, 2) educating health care professionals, patients and payers on the latest innovations in P&O and 3) being held to the highest ethical and moral principles in accordance with corporate compliance and quality assurance plans. The practice was built upon some unique propositions for patients and payer sources that include a lean patient care delivery model focused on utilizing an electronic health records system (EHR), routinely measuring patient outcomes, utilizing best-in-class outsourced manufacturing, and conducting clinical research. With these practice attributes in place, I intended to move the profession from a very ‘device-centric’ focus to a more ‘patient-centric’ experience focus, with emphasis on consistent and repeatable care processes that producing measurable and meaningful changes in patients' functional performances. It was my vision to pursue advancing Ability's mission through organic growth.

By late 2007, Ability had successfully opened four P&O facilities. This gained the attention of an article titled, Conceive-Ability: A New Model for an Old Practice,^2^ which highlighted the ergonomically designed offices to account for the needs of the patient population, as well as practitioner and staff clinical workflow instead of fabrication processes. It was paramount to the practice model that the physical layout and build-out of the facilities be consistent across locations. This agile facility design concept and standardized workflow processes allowed rapid adoption of emerging technologies across the organization. Much like a contract research organization (CRO) would function for a pharma company, we could offer the same value adds for manufacturer and academic research by facilitating access to patient populations.^3^ This same uniformity in physical and operational design and consistency attracted opportunities to conduct clinical research. Ability started conducting clinical research through sub-awards to government funded research grants with universities and technology developers.

By 2010, Ability was a self-proclaimed ‘super user’ of our EHR. The operational consistency and efficiency allowed Ability to expand to five practice locations, with plans for a sixth. The practice model continued leveraging outsourced manufacturing with growing success. However, one limitation to the practice model was variability and non-standard approaches through which payers requested ‘letters of medical necessity’ (LMN) and additional justification for the recommended components. By this point, the gap separating P&O technology advancements and the willingness or ability for third party payers to provide reimbursement for these interventions had grown into a wide chasm. In response, Ability began to develop and submit extensive treatment plans to insurance companies on behalf of the patient to educate the payers and secure authorization. The treatment plans included thorough patient history and evaluation, baseline outcome measures, often the results of trial fittings comparing function of two potential components, images, video, and physical/occupational therapy plans. Additionally, the treatment plans included citations to the latest research publications, which rooted the treatment plans in evidence. The treatment plans were reviewed and countersigned by the referring physician, therapist and often a case manager. Ability also started routinely visiting and meeting with third party payers as frequently as possible. The intention of these meetings was not only to share new technology and treatment protocols. Moreover, these meetings served to keep payers informed of changes within the profession, such as the changing educational requirements that were leading to Master's Degrees for all P&O educational programs by 2012.^4^

While the educational requirements and research evidence in P&O were advancing, the landscape changed again as the Department of Health & Human Services' Office of Inspector General (OIG) released a scathing but refuted report in 2011 entitled Questionable Billing by Suppliers of Lower-limb Prostheses.^5^ This precipitated at least a decade long trend towards various audits, lengthy appeal processes as well as severely limited L-code assignments for new P&O products. The 2011 OIG report and subsequent negative pressure caused Ability and others to redouble efforts to elevate the P&O profession.

**Figure 1:**
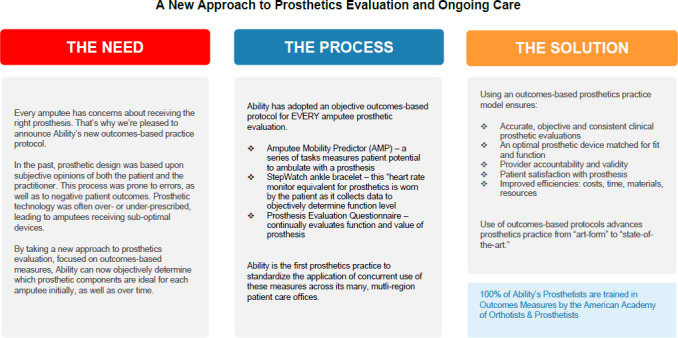
Ability's Lower Limb Prosthetics (LLP) outcome measure protocol.

**Figure 2:**
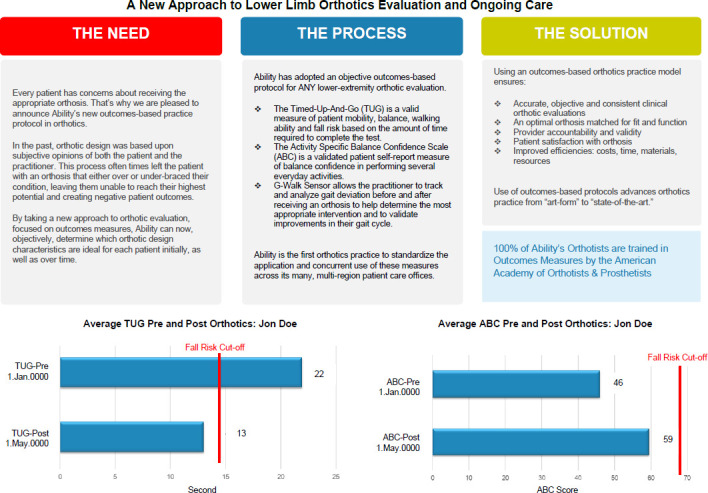
Ability's Lower Limb Orthotics (LLO) outcome measure protocol with a sample Pre and Post Outcome Measure.

In response to demands for improved justification, Ability launched its first formal outcome measure protocol to begin collecting objective patient-centric data to inform treatment recommendations and the component selection process. Outcome measures allowed Ability to document patients' capacities and limitations, improving defense against denied claims and audits. Simultaneously, the outcome measure results advanced the clinical judgement of Ability clinicians through interpretation of patient outcome measures and comparison against normative population data. For example, Ability began by administering the Amputee Mobility Predictor (AMPRO™ and AMPNOPRO™),^6^ the Prosthesis Evaluation Questionnaire – Mobility Subscale (PEQ-MS) and the Socket Comfort Scale (SCS) for patients receiving lower limb prostheses (**Figure1**); the Timed-up-and-Go (TUG)^7^ and Activities Specific Balance Confidence Scale (ABC)^8^ for patients receiving lower limb orthoses (**Figure 2**); and the Disabilities of the Arm Shoulder and Hand (DASH) and Patient Specific Functional Scale (PSFS) to patients receiving upper limb prostheses Ability's Outcomes & Research Director was committed to consistent administration of the outcome measure protocols and also analyzing the data in meaningful ways to benefit both the patient and practitioner.

**Figure 3:**
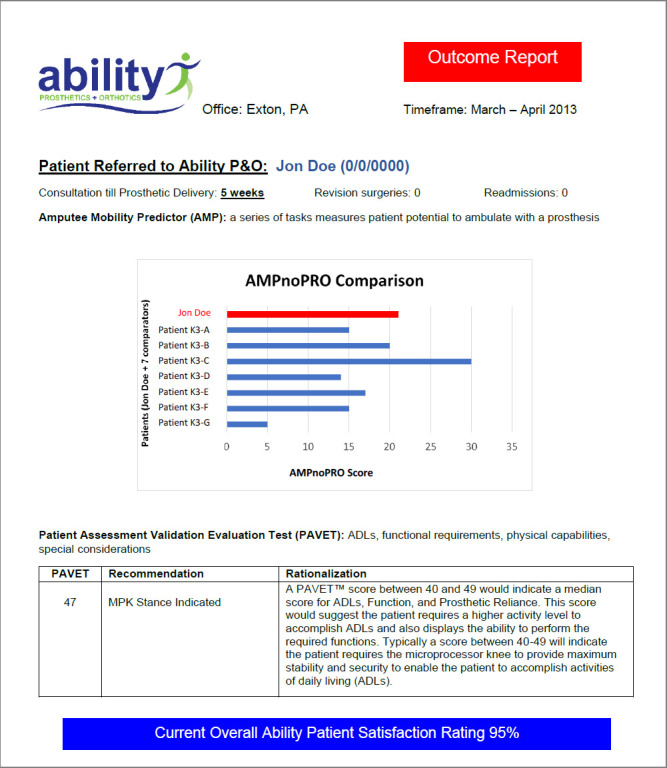
Example of Ability's ‘Early Years’ individual patient outcome measure reports, with summary of delivery timeline and revisions/readmissions, AMPnoPRO results presented in graphic form with seven other K3 (randomized, anonymous) patients for comparison, PAVET score interpretation, and overall satisfaction survey rating, based on an in-house standardized, satisfaction survey.

Ability's thought leadership around outcome measures gained attention within the profession and was captured in an editorial article from 2014 titled, Measuring the Usefulness of Outcome Measures.^9^ The article detailed the approach of using objective outcome measures, documented through Outcome Reports, to inform important clinical decisions, such as assigning a Medicare Functional Classification Level (MFCL) (**Figure 3**). Later on, the data across the entire practice could be queried to evaluate trends across large patient populations to verify the validity and utility of the outcome measure protocols and to gain knowledge about clinical practice not otherwise possible. A digital platform and automated outcome measure report generator was developed to streamline process of collection, interpretation, and sharing of patient outcome measure results with healthcare partners. Digital platforms provide the flexibility of allowing results to be presented in different formats (raw data, tables, graphs) depending on the use and audience for the information.

Ability includes data visualization and automated score interpretation logic in our digital solution. Data aggregation is done on an as-needed basis. This work led to a peer-reviewed journal publication of a retrospective chart review study, a ‘first’ for Ability's blossoming clinical research program.^10^ The results from that publication supported that both the AMP and the PEQ-MS showed promise for assigning MFCL by stratifying patients' capacity and self-rated mobility. Impactful publications, such as this, resulted in more widespread adoption of outcome measures throughout the profession.

This development of Ability's practice model coincided with a concerted progress within the P&O profession toward Evidence-Based Care (EBC). Subjective assessments and ad-hoc or unvalidated evaluation forms designed to satisfy supplier requirements, such as the PAVET,^11^ were replaced with objective, valid, and reliable instruments. This new data source posed an opportunity to evaluate the patient experience and examine the benefit of various products and technology in a real-world clinical setting, as opposed to testing with small sample sizes conducted in manufacture or institutional labs. Ability soon sought opportunities to conduct Institutional Review Board (IRB) approved comparative effectiveness research protocols to evaluate new P&O products.

By 2015, the profession would be faced with perhaps one of its toughest challenges yet, a proposed draft by Health and Human Services (HHS) for a new Local Coverage Determination (LCD) policy regarding requirements for Medicare beneficiaries to qualify for prosthesis coverage.^12^

Among others, a change to the definitions of the MFCL K-levels within the LCD policy posed the gravest threat to patient access to prosthetic technology. Ability's nearly two years of amputee outcome data provided quantifiable evidence of the detrimental effects of the draft LCD, and a report was submitted by Ability during an open comment period to protest the proposed changes. After nearly three months of petitioning the changes, the profession was able to reverse the proposed draft LCD and HHS agreed to assemble an interagency workgroup to further assess the need for refined medical necessity policy and the current state of evidence in the profession.^13^

This moment represented a turning point within the company culture at Ability, as practitioners had a ‘front row seat’ in experiencing the value of objective patient clinical outcome data and the ultimate potential impact on healthcare policy. Ability's role in reversing the draft LCD and protecting patient access to the prosthetic technology energized its practitioners and staff to recommit their efforts to diligently collect outcome measures and demonstrate how P&O interventions improve patients' functional performance.

While most in the P&O profession were reeling from the recent existential threats from reimbursement policy and aggressive auditing practices, Ability recognized the need to expand its clinical research capabilities to address the increasing demand for research evidence. With twelve P&O facility locations by this time, Ability had a reliable access to a large patient population for research recruitment. Additionally, the consistent workflow operations across the organization began to attract interest in developing partnerships to conduct clinical research. Ability developed formal research collaborations with universities, medical practices, and technology developers to seek and secure funding to make clinical trial opportunities available throughout its practice footprint. Ability's practitioners and patients both appreciated clinical research opportunities as a ‘value add’ that further differentiated the Ability practice model within the P&O landscape.^14^

One example of the research conducted at Ability is a clinical trial that evaluated patient-reported and performance-based outcome measures in transtibial amputees with a novel microprocessor-controlled ankle component.^15^ The study was sponsored by the manufacturer, Freedom Innovations, and represents one of the largest investigations of that technology class to-date. The study results were accepted for publication in a peer-reviewed journal, and the presentation at a national conference earned the Thranhardt Best Paper Award award.^16^ This success demonstrated that clinical research posed an opportunity for Ability and the P&O profession to contribute to research evidence at the highest level.

## Establishing Best Practices & Achieving Operational Efficiency

By 2017, the practice model had matured, and Ability was a strong regional patient care provider and healthcare company. Navigating the P&O environment and healthcare landscape was not done without challenge. Ability expanded office locations at times and closed or sold locations at other times. Ability had acquired a P&O practice and integrated it into the practice model. The organization endured several cashflow crunches, outlasted the 2008 economic recession, reacted to countless Medicare policy changes and continues to innovate in the face of the global COVID-19 pandemic.

Through this phase, Ability continued to make significant steps to build the executive management team to include a full C-suite (i.e Chief Operating Officer, Chief Financial Officer, Chief Information Officer, and Chief Compliance Officer), along with Clinical Regional Directors and a Clinical Outcomes & Research Director. The onboarding process was refined to better prepare new hires to excel in the practice model. The residency program was expanded, as Ability was now attracting residents and board-eligible clinicians with master's level degrees. A management scorecard was developed to include both clinical and financial Key Performance Indicators (KPIs) to more closely align patient, practitioner and management on the shared goal of advancing patient care. This investment in organizational and management structure was necessary to support the financial success of an expanding practice model and to provide a dimension of visibility and accountability that Ability's patient care facilities and practitioners were supported in fulfilling the company's mission.

The company invested in and led a branding refresh to update the image of the organization. Ability assembled a Patient Advocacy Council (PAC) from the patients it serves to provide guidance and raise awareness of key patient issues, concerns, and opportunities for the organization. Practitioners and the PAC collaborated in evaluating the patient journey and the care processes that contribute the greatest value to the patient experience. Furthermore, it's been my experience that our profession could also benefit from a similar and evidence-based professional approach to ‘onboarding’ new and existing staff by focusing on communication skills in P&O.^17^ These steps in the process were memorialized as Ability's ‘Patient Care Pathway’. Clinical Best Practice (BP) procedures and training manuals were developed to expand upon and provide actionable guidance for each step in the Patient Care Pathway. The Best Practice manual was implemented in the onboarding process and referenced by experienced practitioners to support repeatable delivery of the most vital patient care processes. This activity and the resulting Patient Care Pathway and Best Practice manual were vital in capturing the shared clinical experience and ‘tribal knowledge’ of the organization in a tangible and useful form. It's been my personal experience, practices historically onboard practitioners with ‘tribal knowledge’, rather than taking a more formal, repeatable approach.

By the beginning of 2018 and still today, the organization had made significant progress internally through documenting Best Practices and continuously improving clinical processes; including our ability to collect, interrupt and report patient data as illustrated with our most recent outcome report. Ability was on solid financial ground, but any P&O practice could always stand to improve the balance sheet. By this time, I had transitioned away from much of the ‘day to day’ operations of the organization and was focusing more on business development initiatives, ‘culture mining’, and coaching others within the organization. Ability reached a certain operational maturity across the organization for the first time with the executive management team fully in place. Timing was good to explore how Ability might extend the practice model to reach more patients. Exploring opportunities with a partner on a larger scale emerged as a potential approach to future growth.

Around this time, I also had the opportunity to serve on the American Orthotic & Prosthetic Association's (AOPA)^18^ board as well as on an executive panel for the National Limb Loss & Preservation Registry.^19^ These roles provided me with a new perspective from the ten-thousand-foot view of the entire P&O profession and also from a glimpse of how the rest of the healthcare sector views the P&O profession. These unique experiences influenced my beliefs about the role and impact that Ability could have in re-defining the care delivery model and advancing the standard of patient care. It became apparent that to achieve the full potential of the practice model and to reach the goals the organization set out to achieve, Ability would be best served by collaborating with like-minded P&O practices in a nationwide US network with a strategic partner. Such a partnership could allow a collective response to external forces within and external to the P&O profession and a concentration of resources and expertise that individual organizations would not otherwise be able to harness.

## Finding A Strategic Partner

So how does one go about finding a strategic growth partner? Ability's board of directors recommended following a simple principle: Look for strategy, financial and cultural alignment that will help identify a solid foundation from which to pursue growth in a true partnership.

I believed that the most likely option for alignment on culture and mission, with the best opportunities for accelerated and sustainable growth, was a strategic partnership with an organization within the P&O industry. While the allure of private equity (PE) backed funding can be exciting, I don't believe the P&O profession can provide the expansion expectations and sought-after return on investment in a short enough timeline to satisfy the goals of PE groups. Other hybrid style lenders do exist, who could entertain moderate growth plans. However, those financial lending services require more debt. Partnering with an organization within the P&O industry matched the recommendation from Ability's board of directors best.

Ability had been approached by and had several meetings with other large P&O practices over the recent years. We had opportunities to become a part of a larger P&O practice organization, but that alternative always seemed to stifle the organizations entrepreneurial spirit and limit the influence of Ability's unique practice model. When manufacturers within the P&O industry began to show a definitive interest in strategic partnerships with direct patient care practices in the US, it became very clear how a strategic partnership with a manufacturer presented several synergies. Ability could continue the thoughtful growth trajectory that the organization has enjoyed. Ability's best-in-class clinical practice platform could assist in launching a venture into patient care, and the clinical research and outcome measure competencies of the organization could be additive to improving the products and services available to the P&O profession and patients it serves.

**Figure 4:**
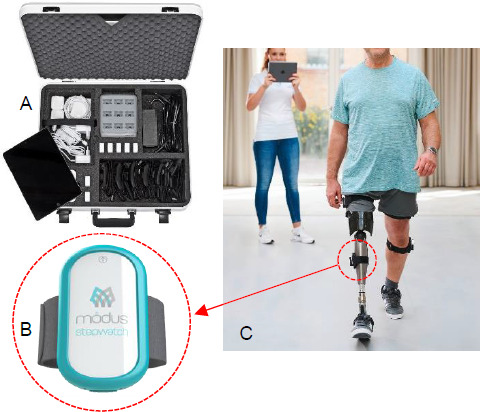
Real real-time sensors to aid in data capture, developed specifically for orthotics and prosthetics are used at Ability as part of daily clinical practice. **A**: Ottobock Bionic Pro; **B**:ModusTM StepWatch; **C**: Real-time, untethered gait analysis being carried out using the Bionic Pro and StepWatch. Persons in image have given informed consent to publication.

In early 2020, Ability consummated an investment from the world's largest prosthetics manufacturer, Ottobock Healthcare. Ability became a cornerstone in the newly formed business unit, Ottobock Patient Care. Together, we can now advance patient care standards in the US by first working to strengthen our practitioners use of evidence to make evidence-based and data-informed clinical decisions regarding treatment plan and component recommendation. We can leverage Ottobock's vast experience in P&O patient care around the world, product technologies, clinical research and reimbursement experience, growing amputee data lakes and existing Best Practices in the areas of lean facility design, scanning and fabrication, as well as quality assurance. We can accelerate data capture through real-time sensors and further automatic current processes, thereby bringing meaningful translation of the data; to bring those ‘daily practice’ efficiency gains to the practitioner, saving them time and providing a more individualized experience to the patient. (**Figure 4**)

## Call To Action

As P&O patient care providers, we must learn how to maximize digital options, including automation and artificial intelligence to create local clinic practitioner efficiencies.

We must have access to meaningful and actionable data that supports risk-adjusted, evidence-based treatments and quality improvement that will be paramount in providing individually optimized patient care-which in itself further defines our value proposition to patients and payers alike in which to continue to advance the profession.

I would call for more manufacturer sponsored, IRB-approved clinical research during product development cycles. We should also use the translation of the data we collect to spur additional research as well as to inform governmental and commercial payer P&O policy changes.

I would call for changes in the MSPO curriculums to include the teachings of Health System Science.^20^ This will better prepare the clinical leaders of tomorrow to become better interrupters with meaningful patient data to inform their patients, payers and healthcare team members. This will also help to develop the clinical leaders we'll need in an evolving healthcare delivery system.^21^

We will need to demonstrate and publish frequent progress on the above-mentioned fronts to further advance comprehensive P&O legislation that sanctifies what we do as clinical care providers- not as device-centric and off-the-shelf brace suppliers. That will allow us to use this progress to help define a collaborative scope with Medicare around competitively bid commodity products vs. the value of evidence based clinical care. We should consider transitioning P&O practitioners to the healthcare provider status in Medicare's eyes with prescribing capabilities. A big challenge will be to continue to update the L-code reimbursement system to assimilate newer technologies at an equitable reimbursement rate in a timelier manner. We should advance concepts that support the transition to a fee-for-value (FFV) reimbursement structure and work to adapt, support and inform PDAC verification changes that actively affect our profession.

## DECLARATION OF CONFLICTING INTERESTS

The author is the founder, employee and a shareholder of Ability Prosthetics and Orthotics, Inc. The author is an employee and shareholder of Ottobock Patient Care. The author is a business advisor and a shareholder of Impulse Technology, LLC.

## SOURCES OF SUPPORT

Industry funding was provided by Freedom Innovations to carry out a clinical trial that evaluated patient-reported and performance-based outcome measures in transtibial amputees referred to in this paper.

## AUTHOR SCIENTIFIC BIOGRAPHY

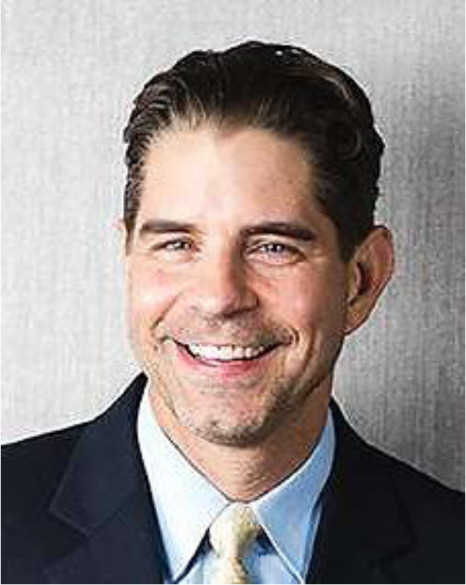



**Jeffrey M. Brandt, CPO,** Chairman & Founder, Ability Prosthetics & Orthotics; Director, Business Development, Ottobock Patient Care.

**Jeffrey M. Brandt,** founded Ability Prosthetics and Orthotics, an evidence-based P&O practice that has grown to twelve offices across three states. Since its inception in 2004, Ability has led the advancement and implementation of outcome measures in daily practice, founded Lifenhanced magazine, and assembled a patient advisory council to inform of patient-centric best practices. Through Brandt's leadership, Ability has prioritized a focus on patient care, outcomes data, delivery of care, business analytics, community based adaptive programs and comparative effectiveness product research and development. In addition to founding Ability, Brandt is a co-founder of Kinetic Revolutions of which the most notable product is the height adjustable pylon for use on prosthetic limbs. After graduating from Penn State University in 1995, Brandt completed the prosthetics technician program at Spokane Falls Community College in Spokane, Washington and became an ABC certified technician. He then attended Northwestern University's Feinberg School of Medicine's Prosthetic & Orthotic program in 1999. He subsequently completed his orthotic residency at the Rehabilitation Institute of Chicago and his prosthetic residency at Lawall P&O in Delaware, where he serviced A.I. DuPont Children's Hospital. As a student, Brandt was awarded the Gunther Gehl Prosthetics Scholarship by the Midwest Chapter of the American Academy of Orthotists and Prosthetists (AAOP) and has been named to the O&P News 175. Brandt currently serves on the American Orthotic & Prosthetic Association's (AOPA) board, Limb Loss & Preservation Registry External Collaborative Panel (ECP) and as a business advisor to Impulse Technology.
